# Benefits and harms of placebo in surgical randomised clinical trials: a systematic review

**DOI:** 10.1186/1745-6215-14-S1-P39

**Published:** 2013-11-29

**Authors:** Karolina Wartolowska, Andrew Judge, Benjamin Dean, Ines Rombach, Julian Savulescu, David Beard, Andrew Carr

**Affiliations:** 1 Nuffield Department Nuffield Department of Orthopaedics, Rheumatology and Musculoskeletal Sciences, Oxford, UK; 2 Oxford Uehiro Centre for Practical Ethics, University of Oxford, Oxford, UK; 3 MRC Lifecourse Epidemiology Unit, Southampton, UK

## Background

Placebo-controlled randomised clinical trials on surgical procedures are rare and there are concerns about the safety of the placebo arm. The aim of our study was to analyse the balance between benefits and harms in placebo-controlled surgical trials.

## Methods

We searched MEDLINE, EMBASE, and the Cochrane Controlled Trials Register, from their beginning to September 2012, and screened the references of the already included studies. We reviewed randomised clinical trials comparing surgery with a placebo intervention. Surgery was defined as any procedure that changes the anatomy and requires a skin incision or the use of endoscopic techniques; dental studies were excluded. We did not limit the search to any particular type of intervention or condition.

## Results

We reviewed 52 good quality studies. The incidence of adverse events was higher in the active group and many of the complications were related to the investigated critical element of the surgery. Harms directly associated with the surgical placebo occurred in 4/52 studies, in 13/52 adverse events in the placebo group were related mainly to the condition and not the intervention, and in one further trial to both factors. There were no serious harms arising from the use of a general anaesthetic.

## Conclusions

There is a need for more surgical randomised trials with a placebo arm. Without studies like this there is a risk of substantially increasing the use of useless and sometimes also harmful procedures. There is a need to “de-stigmatise” and extend the use of the surgical placebo in clinical trials.

